# Implantation Surgically Considered

**Published:** 1889-06

**Authors:** Rodrigues Ottolengui

**Affiliations:** New York


### [1] IMPLANTATION SURGICALLY CONSIDERED.

RODRIGUES OTTOLENGUI, M.D.S., NEW YORK.

Whether the operation of implantation be destined to abandonment when we are enabled, if ever, to form a final judgment, or whether it will become a recognized practice, is not here to be discussed. In either case, it is however of much importance at present to those who undertake it, to possess a thorough knowledge of the anatomy of the maxillæ. To the inexperienced, who attempt to operate without such knowledge, there is a danger which cannot be overestimated. It is to be borne in mind that vessels are only approximately constant in their positions in the body. The surgeon cutting through soft tissues, is warned by his sense of touch, if he approaches a vessel slightly out of the expected position. With us, who drill into a bone, however, the conditions are different. In the superior maxilla the vessels do not pass through special channels, but readily penetrate the spongy bone. In the inferior maxilla where the vessels are larger, there is, except in the incisive region, a special canal. This canal, however, has no dense wall which might give us warning, but is formed simply by a slight condensation of the bone, and retains in a marked degree the spongy appearance. The positions of the nares and antrum should be thoroughly appreciated. In order to gain a full insight into these matters, I obtained a number of bones, made sections in various directions, and what I learned from these and actual operations in the mouth, are the sources from which I draw the conclusions arrived at in this paper.

---

[1] Read before the Odontological Society of Pennsylvania, February 2nd, 1889.

In an article on Replantation in the American System (Vol. II, page 372), Dr. Weld makes this statement: "Some practitioners have been in the habit of washing out the socket, and immersing the tooth in a solution of carbolic acid. There is no objection to this plan, provided the solution be not too strong, not exceeding 4 or 5 per cent. *But as antiseptic measures are hardly required in such operations, there is no particular reason for commending them.*" I introduce this here without further comment than to say that Dr. Weld, at one time an enthusiast on the subject of replanting roots and teeth, finally reported failure in a large majority of his cases, after a time varying from a few months to five or six years.

It is my purpose to consider the subject in strict adherence to its title, and I shall describe the operation as I deem it should be performed.

The patient having consented to the operation, the surgeon should first thoroughly wash his hands and clean his nails. I deem this of so much importance that I shall tell how to do it. A basin of water should have about three drops of ammonia added, and the hands should be steeped in this for at least one minute, and then thoroughly scrubbed with a hard brush, using castile or glycerine soap. The hands thus relieved of the main portion of stains and dirt, and the nails cleansed whilst still softened by the warm water, should nevertheless undergo a second washing and scrubbing exactly similar to the first, except that a hot solution of hydronapthol and water should be substituted for the ammonia water. Anyone who has not given the hands a treatment similar to this will be surprised and pleased at the result. In drying the hands a fresh clean towel should be used. Of course the assistant should pursue the same method. Next, as to instruments. It is not necessary or advisable to place expensive instruments within the destructive influence of bichloride of mercury. A glass finger-bowl may be partly filled with hot water, and into this a tablet of hydronapthol thrown, producing a saturated solution (1–300), and into this all the instruments to be used should be placed and kept during the procedure, without fear, as this agent is germicidal in a solution one-tenth as powerful, and is non-corrosive. Into the same bowl I place small pieces of sponge, which I use for cleaning the socket of bone chips, a fresh piece being taken each time. In passing, I would suggest that it would be well for the bowl to be of a deep blue or red color, as the instruments thrown in during the operation dye the water, and render it unsightly to a patient of delicate nerves. For the same reason a spittoon should be used which conceals its contents, and should be cleansed as soon as the actual operation is over, that neither the sight nor the odor of the blood may be present. The napkins should be renewed frequently, clean ones replacing those which become stained. Strict attention to these seemingly trifling details does much to mitigate the shock of the patient.

As an anesthetic before making the incision through the gums, I use an ether spray. To those who at this moment are thinking of "sloughing" as an attendant evil of this method, I will say I have used the spray for minor operations about the mouth, opening abscesses, removing necrosed tissues, etc., and have never seen any ill result. I think this due to the fact that the spray is not pressed to the point where actual freezing occurs. With the Rollins tubular knife a circular incision is quickly and painlessly made, and the tissue thus isolated is dissected away with a bistoury. Next, the the socket must be formed. For this no anesthetic is needed. I use instruments of my own pattern for this purpose. I have ten different sizes, which enable me to choose with reference to the diameter of the root of the tooth to be inserted. Two sizes will be found necessary, the smaller, to drill the socket the required depth, and a larger, to ream the upper portion to fit the root. If the larger reamer is a trifle smaller than the diameter at the tooth-neck, the socket may be formed so that the tooth when placed will fit tightly. I have so placed teeth, and seen them worn with comfort, and become fixed without splint or ligature. It is safer, however, to use a splint or retaining fixture of some kind, as the teeth frequently loosen in a few days, though firmly placed at the time of the operation.

Special precautions are to be observed in forming the sockets in every case, as follows:

### First—The Central Incisors, Superior.

A section through the center of the socket of the central incisor in the upper bone shows that the floor of the nares (the inferior meatus), is not far distant, whilst just back of the palatal wall of the socket, and towards the median line is found a canal of considerable size—the anterior palatal canal. Danger of entering the nares is present in two conditions: First, where a tooth, whose root is too long, is used; and second, where there has been considerable absorption after extraction. In choosing teeth, the fact that a tooth in the hand has a crown which matches in size the similar tooth still in the mouth, should, by no means, be taken as evidence that the length of the roots are equal. In development of teeth, the length of their roots is in relation to the depth of the alveolar processes, whereas the width and length of the crowns may more probably depend on the space they must occupy in the arch. Hereditary influences also play an important part. The length of a root cannot be guessed at by the crown. In determining, then, the length, which would be safe, several points may be taken into consideration: First, observe the gum as to whether recession has occurred. If so, a tooth with a slightly longer crown than that of the one next the space, must be chosen, and the root should be just so much shorter. If the lip is turned up, a digital examination will frequently disclose the end of the neighboring tooth and measurement made. If this cannot be done, the line of union between the lip and gums may be considered a point beyond which it is unsafe to drill. In relation to the palatal canal, the direction is an angle slightly greater than the slant of the centrals in position. Generally these two canals are quite close together, or united at the junction of the two bones; but in some specimens, especially from young persons, they are larger and more widely separated. There is a condition frequently seen where a space exists between the central incisors, the frænum continuing down on the gum as a well-marked ridge, ending in a little pedicle, which drops between the teeth. If one or both of these teeth should be lost, and the patient desires implantation, no attempt should be made to fill the space by making the socket (or sockets) near the median line. Such a course would surely result in entering the canal and rupturing the vessels, for the canals are widely separated in this somewhat abnormal condition. Great care should be observed with children —I have inserted a tooth for a boy ten years old to replace one lost by an accident —because the canals are very large at the age of five, being about double the adult. I have seen a skull, with the tempory teeth still *in situ*, where the foramina of these canals were three sixteenths of an inch in diameter. The mere sight of that bone would make an operator redouble his precautions in future operations.

### SECOND—THE LATERAL INCISOR.

In drilling a socket for a lateral incisor, the operation is in less dangerous territory, the distance to the nares being greater, and no large vessels being near. There is, however, one point which it is well to remember, viz., that extreme care should be taken not to drill through the alveolus at the extremity of the socket at the palatal aspect, thus wounding the soft tissue which covers the roof of the mouth. Of course this is an accident to be avoided in all instances, yet I think there is special need for caution with this particular tooth, because whilst I have no proof I have abundant evidence which tends to indicate that an idiopathic tendency to pathological disturbance exists in this specific region. This of course is dogmatic, but caution is never hurtful. In the absence of direct proof, I will simply ask if the gentlemen present have not noticed the more than proportionate frequency of alveolar abscesses in connection with this tooth, and the stubborn persistency of the disturbance when once present; the rarity of success in efforts to conserve even slightly exposed pulps, and the rapid inflammatory action and loosening of the teeth after the death of the pulp? In my own experience I have seven times observed a detachment of the soft tissues from the palate process by the burrowing of pus, in three instances in defiance of the fact that fistulæ existed at the labial aspect of the alveolus. In another instance, almost the entire palatal process was destroyed as far as the median line, by solution of the lime salts from the bone. And in yet another a similar condition of less extent was reported by a brother dentist, in which case apparent restoration to health would result from treatment, the discharge of pus recurring persistently, however, for ten months at each menstrual period. It may be entirely a coincidence, but these nine cases were all in connection with the lateral incisors.

THIRD—THE CUSPIDS.

The cuspid socket normally is immediately under the apex of a triangle of which the wall of the antrum forms one side and that of the nares the other. The distance between the socket and the roof of the mouth is also greater here. A glance at sections of the bone, whether made through the socket towards the palate, or through all the sockets of the bone, shows plainly that this is the safest region for the operation. A point of interest, however, not to be overlooked is the fact that in this territory only do we find vessels of sufficient size to require special canals. I have found canals, two in number immediately over the apex of this socket, and nowhere else in the bone. They are short, however, and cannot be followed far with a bristle. The nerve comes down from the region of the antrum, and immediately over this tooth divides into anterior and posterior dental. In selecting teeth for this part of the mouth we should not choose too long a root, for in making a socket sufficiently deep we might wound one of these nerves, as I am sure I did on one occasion, producing a neuralgia which persisted twenty-four hours, and disappeared only on the exhibition of antipyrine in large doses. An arteriole might be met here, which if injured would, in one of hemorrhagic diathesis, produce unpleasant, if not serious results.

FOURTH—THE BICUSPIDS.

These teeth may be considered together, for the antrum, the point of danger, is sufficiently near in many cases even to the first bicuspid, to make the precaution which should be observed in making sockets for the second bicuspid equally necessary in cases where the operation is for the first. The bicuspids are the teeth we are most frequently called upon to insert, and are also the ones offering the greatest danger of accident. It frequently occurs that, though the adjoining teeth are still in position, the absorption of the alveolus is so great that if the original tooth could be found and replaced, a considerable portion of the neck of the tooth would be exposed. When it is remembered that this loss of tissue shortens the distance to the antrum, originally none too great, the selection of a tooth to occupy this place becomes an important consideration requiring mature judgment. As it is claimed to be impossible to judge of the root of a tooth by its crown, so, also, is it equally difficult to locate exactly an antrum by examination of different skulls. The cavity is most variable in size, being sometimes large in small bones and small in large bones. There is, however, a comparatively safe course to take in the diagnosis, and that is, that the antrum has the largest possible dimensions. This would mean that the floor extended over the entire molar and bicuspid region, the lowest point being over the second bicuspid. The landmarks and their value are as follows: The junction of the cheek and the gum is usually on a level with the floor of the antrum. The molar process, easily discoverable by a digital exploration, forms one floor of the antrum, though not the lowest; the extreme base of this prominence may be taken as a point beyond which it would be unsafe to drill, and measurement may be made accordingly.

In performing the operation the patient should be tipped back so that the height of the roof of the mouth may be well considered, as also the width of the process; this latter point will give an idea as to how much towards the palate the drilling instrument may be deflected in order to avoid the antrum, which is mainly in the buccal portion of the bone, but in this effort to avoid one danger another should not be overlooked; in so slanting the instrument there is a chance of entering the nares, especially in the case of the first bicuspid. It should be borne in mind that should either of these cavities be entered and a tooth implanted notwithstanding, as has been advised by good operators, diagnosis would be difficult should any after-trouble occur. In either case a discharge would find its way into the fauces, dripping from behind the velum. One more point, and then I shall relate an incident which will emphasize the fact that these precautions are worthy of note. After a tooth is extracted, the socket is usually filled with a deposit of new bone. It becomes a question of importance whether this bone be dense or not. In some, and perhaps the majority of cases, the new bone is about the same as the surrounding parts. In other cases, however, and these are the ones fraught with special danger, when working under an antrum, the socket is filled with bone abnormally vascular in character, the surface, however, being a dense, hard layer. In operating in such a case, the outer and denser layer would offer such resistance that great force would be required to penetrate it, and this accomplished, were the operator not prepared against accident, the instrument would rush forward through the less resistant spongy portion. In order to exemplify this, and to disprove the assertion made by a skillful operator in a paper read last summer, to the effect that no serious consequences might be expected from entering the antrum, I will insert here the history of a case in which occurred most untoward results.

At the clinic before the First District Society of New York, last winter, I implanted a first bicuspid for a lady patient. I found the bone at the surface very resistant. I was using the Walker-Younger trephine, and had the little collar set to indicate the depth to which I had decided it would be safe to drill. I wish to emphasize the fact. that it was for a *first* biscupid for which I was preparing a socket. I exerted considerable pressure in entering the bone and was, nevertheless, progressing slowly, when suddenly, to my amazemet and dismay, my instrument plunged forward, entering the bone beyond the depth marked by my collar, and became so wedged that my engine was stopped. I had considerable difficulty in withdrawing the trephine, and a flood of blood followed. A spittoon was furnished, but my patient continued ejecting blood so long, that I brought her head back into position and attempted to wash the parts for an examination. This was done with difficulty, as the flow of blood was quite continuous. At last, however, it was staunched sufficiently by packing the cavity with cotton, so that the mouth and face could be washed, and then, on withdrawing the packing, as the flow began again, the nature of it was determinable, and from the distinct pumping, I concluded that it came from an arteriole. I again packed the cavity, as the flow threatened to be great, and with a careless word to those about me, none of whom suspected the gravity of the situation, knowing neither that the antrum had possibly been entered, nor that a vessel had been lacerated, I whispered a word of encouragement to my patient, and turned away to seek advice from friends present. One of great experience returned with me, and watched the flow as I removed the packing a second time. He advised the rapid completion ot the operation and setting of the tooth in spite of the hemorrhage, which was really alarming. In deference to this advice, I finished my operation rapidly and placed the tooth in position, procuring a fine adaption and perfect occlusion, and winning encomiums from all present, except myself. I received the applause accorded me with a heavy heart and a sore conscience. Fortunately for me, the patient was my own sister, and would be under my constant observation. Before placing the tooth finally, I essayed to probe the socket, in order to determine whether the antrum had really been penetrated. I could find no opening, but touched a point which was so very sensative, that I had little doubt that it was a nerve. The exploration also brought a recurrence of the hemorrhage, and for this reason was not thorough.

The first night an increasing pain was present, reaching such a point by midnight, that I deemed it best to administer twenty grains of chloral, which, from an acquaintance with the idiosyncracies of the patient, I knew to be the safest remedy. This brought sleep in about one hour, and she reported at my office the following afternoon. At that time the pain still persisted, and she complained of a slight but constant trickling of blood in the throat. An odor of blood also began to be noticeable. That night sleep was obtained without recourse to narcotics, and my own mind became somewhat relieved.

On the third day the patient reported at my office in the morning. Locally all seemed progressing well; but the dropping of clotted blood from behind the soft palate was still present, and the odor had so much increased that a spray of carbolized water was used, and directions given that it be kept up during the day. The neuralgic pain from the lacerated nerve still continued, and antipyrine was ordered in doses of five grains every hour. As the patient seemed cheerful, I kept an engagement previously made for that evening, and did not reach my home in Brooklyn till midnight.

I found my sister awake and anxiously awaiting me. The dripping into the fauces had become purulent to a slight degree, and though the antiseptic spray had been strictly adhered to during the day, the oral cavity emitted an odor disgusting even to the patient. She was in a high fever, the temperature being 102°, and the pulse 100, the normal pulse in her case being as low as 70. This fever continued, notwithstanding the administration of the antipyrine, which had produced no salutary effect whatever, the pain having greatly increased. Under the circumstances I deemed it best to remove the tooth, which was done gently with the fingers. The immediate result was a great intensification of the pain; but chloral produced sleep in about three quarters of an hour.

We arose about seven o'clock the next day, and called on a throat specialist. My intention in so doing was to have him make an examination, in order if possible to discover whether the dripping into the throat came from the antrum or the nares. I remembered that, in order to accommodate the occlusion, I had slanted my trephine towards the latter cavity. We reached the office of my friend before he had arisen, and whilst awaiting him, my sister lay on the lounge. In about five minutes she arose, saying that blood was coming from the *socket.* I at once took her into the office, and sent a messenger to hasten the doctor, as I knew not where he kept his appliances. This secondary hemorrhage assumed the most alarming proportions, the flow being continuous for half an hour, in spite of our efforts to control it, which was finally accomplished by packing the socket and then forming a roll of cotton on which she could bite, thus making a compress. The actual flow having ceased, we allowed her to rest on a lounge, whilst the doctor attended to his patients, who began to arrive. For two hours, in spite of the packing and the compress, sufficient blood oozed through the dressings so as to make the presence of a spittoon necessary.

At the end of three hours we returned to the specialist's office. Cocaine was applied to the nose and the cavity dilated. A thorough examination was made, both ocularly and with instruments; but no evidence that the discharge came from the antrum was obtainable. Instruments wrapped with cotton could be passed through the passage and withdrawn free from any stain of blood. So long as the hemorrhage had continued the dripping into the throat had ceased, as also had the pain; both had recurred with the packing of the cavity. All the unpleasant symptoms continued during the day. The spray was changed to one of listerine, as being less dangerous to the mucous membrane; the packing in the socket was left in place, and specially sprayed to keep down the offensive odor.

On the morning of the sixth day (two days later), the patient awoke to find that the packing had slipped out of the socket during the night, and as the hemorrhage had not recurred it was left out. That afternoon she was able to come to the office, having been confined to bed for two days, suffering from nervous prostration and pain. As the discharge into the throat was now distinctly pus, in spite of the danger of renewing the hemorrhage, a gold probe was passed into the socket, in the hope of determining whether the antrum was involved. The probe passed into the antrum unobstructed, and all doubt was dispelled. No serious result followed this probing, although slight hemorrhage occurred that night, which the patient herself readily controlled by packing the socket.

The question arose as to what was the best method of procedure, and in this affair I felt myself irresponsible, the nervous strain on myself and sleepless nights having completely unmanned me ; I therefore called in a friend in consultation, and we discussed the propriety of placing a tampon in the opening to the antrum, so that that cavity might be thoroughly washed out. We determined that this would greatly aggravate the pain and delay the healing; that so long as the cavity was being emptied by the dripping behind the soft palate, the danger of septicemia would be absent, and therefore such heroic treatment not indicated. The course decided on was to use antiseptic sprays in the mouth and nares, and wait. If the dripping should cease and the temperature rise coincidently, interference would then become necessary.

This treatment was followed, and a cure effected. The pain, however, continued for fifteen days after the operation, the dripping of pus continuing for thirty days, and a complete restoration occurring in about five weeks.

Undoubtedly the hemorrhagic diathesis in this case contributed greatly to the disastrous results of what, after all, was but a simple wound. There could scarcely be an accusation of septic taint, as the strictest germicidal treatment has always prevailed in my operations. It was only the eighth day after the operation that I was, by appointment, to give a clinic at Newburgh, and my feelings during the operation may be imagined. The case was the implantation of two central incisors, in a space much too wide, and the dentists about me endeavored to persuade me to close up the space at the median line. I had too lately had an experience of hemorrhage, however, to approach the palatal canal, and set the teeth, as the original ones had been, with a space. I think the relation of this case should tend to make operators careful, especially where there is a predisposition to hemorrhage.

FIFTH—THE MOLARS.

Of molars there is little to say. The usual precautions are necessary, especially as to the antrum. I generally use wisdom teeth which have a single root, or convergent roots, or possibly a lower molar, but never a three-rooted tooth. I make a single socket, not a socket for each root.

In the *lower jaw*, all the general and a few special precautions are necessary. The point of danger is the presence of the dental vessels, which occupy a large canal, the locality of which should be specifically known. A section through the centers of the sockets, which removes the lingual portion of the bone, discloses the fact that the dental canal would thus be reached only in the bicuspid region. A section, however, which removes the buccal portion exposes the entire canal. The point of moment to be gathered from this is, that the canal exactly underlies the teeth only in the bicuspid region; in the molar region it curves outward, and occupies a position in the swell of the bone. Between the bicuspids the canal divides, one branch curving outward, reaching the surface at the mental foramen; the other continues, but is no longer a definite canal when the cuspid is passed.

FIRST—THE INCISORS.

The incisive region, in the lower jaw, is *comparatively* safe. It must be remembered, however, that whilst there is little danger to be apprehended from drilling deeply, much care must be exercised in lateral reaming, as the alveolus is narrow, and, here as elsewhere, the aim should be to surround the implanted tooth with bony rather than with soft tissues. The amount of tissue lost from absorption, caused by salivary calculus, should be carefully estimated in deciding the depth to which the drill may be carried. In fact, the aim should always be to carry an artificial socket no deeper than was the original one.

SECOND—CUSPIDS.

Under the cupsids be it remembered, the dental canal, though smaller than elsewhere, is nevertheless distinct.

THIRD—BICUSPIDS.

In connection with these teeth it should be borne in mind that the main canal is immediately beneath the original sockets, and that a branch passes between them. A socket for a first bicupsid should be drilled towards the cupsid, and one for the second should be directed towards the molar. The depth to which the drill may be carried should be most carefully calculated.

FOURTH—MOLARS.

It having been shown that the canal, quite large in this vicinity, curves outward towards the cheek; it would be safer to slant the drilling instrument lingually, rather than straight down or outward.

In passing this aspect of the subject, a few more cautions may be noteworthy. I can conceive of no condition where it would not be reprehensible to endeavor to implant a third molar, especially in the superior jaw where there would be danger of wounding the artery which passes around the condyle, the superior dental, or the descending palatine, within the posterior dental canal.

The arteries which supply the teeth are larger in the lower than in the upper jaw. In the latter, within the bone itself, are the inferior dental which, under the bicuspids divides, the mental passing out at the foramen, and the incisor continuing under the anterior teeth, till it anastomoses with its fellow. In the superior maxilla it is otherwise in that the branches of the superior dental, which enter the foramina of the bone to supply the teeth, are too small to be given distinctive names, but are still large enough to make considerable hemorrhage possible, as has been related. We do not trace back far before we reach important vessels, the superior dental being a branch of the alveolar, which is given off by the internal maxillary, the most important branch of the external carotid.

Absorption in the lower jaw is usually so great, that the original distance between the dental canal and the surface of the bone is materially lessened. Except where but one tooth is absent it would be imprudent to operate without special consideration of this fact. For example, suppose a case where the bicuspids and molars have been lost on one side; the implantation of a molar would render a bridge possible; but where so many teeth had been lost it would be very improbable that sufficient alveolar process would be remaining above the canal to render the operation possible. If such a bone be examined, even the mental foramen will be seen to be almost at the surface.

The socket made, it remains to place the tooth in position. Before this is done, all bone chips should be thoroughly removed, pieces of sponge being used ; one piece saturated with hydronapthol solution being left in the socket, whilst the operator once more thoroughly washes his hands and sterilizes them. In the final placing, the fingers which insert the tooth, and the tooth itself should be wet with the potassio mercuric iodide solution. A piece of orange wood and a few taps of a mallet may be used on occasions to set the teeth home.

I prefer the splint to the ligature for retaining. A permanent splint is made of gold, swedged to form a continuous crown and backing over three teeth including the implanted one. This is fastened with oxy-phosphate. A removable splint is made of vulcanite and covers the roof and passes over the cutting edges of three teeth as before. This worn continuously at first is, after a while, worn only at night, to prevent accident during sleep. In cases where the patient can be seen often the latter splint will be found preferable. Impressions for these are made with plaster immediately on completion of the operation. The plaster is watched closely and removed in such a way as to fracture at the cutting edges, leaving the portion which covers the face of the teeth and the gums, to be removed afterwards. If done carefully there need be no fear of disturbing the tooth implanted.

